# Artificial Intelligence in Inflammatory Bowel Disease Endoscopy

**DOI:** 10.3390/diagnostics15070905

**Published:** 2025-04-01

**Authors:** Sabrina Gloria Giulia Testoni, Guglielmo Albertini Petroni, Maria Laura Annunziata, Giuseppe Dell’Anna, Michele Puricelli, Claudia Delogu, Vito Annese

**Affiliations:** 1 Unit of Gastroenterology and Digestive Endoscopy, Scientific Institute for Research, Hospitalization and Healthcare Policlinico San Donato, Vita-Salute San Raffaele University, San Donato Milanese, 20097 Milan, Italy; 2 Unit of Gastroenterology and Digestive Endoscopy, Scientific Institute for Research, Hospitalization and Healthcare Policlinico San Donato, San Donato Milanese, 20097 Milan, Italy; 3 School of Specialization in Digestive System Diseases, Faculty of Medicine, University of Pavia, 27100 Pavia, Italy

**Keywords:** inflammatory bowel disease, artificial intelligence, endoscopy, endoscopic activity, histological activity, dysplasia detection

## Abstract

Inflammatory bowel diseases (IBDs), comprising Crohn’s disease (CD) and ulcerative colitis (UC), are chronic immune-mediated inflammatory diseases of the gastrointestinal (GI) tract with still-elusive etiopathogeneses and an increasing prevalence worldwide. Despite the growing availability of more advanced therapies in the last two decades, there are still a number of unmet needs. For example, the achievement of mucosal healing has been widely demonstrated as a prognostic marker for better outcomes and a reduced risk of dysplasia and cancer; however, the accuracy of endoscopy is crucial for both this aim and the precise and reproducible evaluation of endoscopic activity and the detection of dysplasia. Artificial intelligence (AI) has drastically altered the field of GI studies and is being extensively applied to medical imaging. The utilization of deep learning and pattern recognition can help the operator optimize image classification and lesion segmentation, detect early mucosal abnormalities, and eventually reveal and uncover novel biomarkers with biologic and prognostic value. The role of AI in endoscopy—and potentially also in histology and imaging in the context of IBD—is still at its initial stages but shows promising characteristics that could lead to a better understanding of the complexity and heterogeneity of IBDs, with potential improvements in patient care and outcomes. The initial experience with AI in IBDs has shown its potential value in the differentiation of UC and CD when there is no ileal involvement, reducing the significant amount of time it takes to review videos of capsule endoscopy and improving the inter- and intra-observer variability in endoscopy reports and scoring. In addition, these initial experiences revealed the ability to predict the histologic score index and the presence of dysplasia. Thus, the purpose of this review was to summarize recent advances regarding the application of AI in IBD endoscopy as there is, indeed, increasing evidence suggesting that the integration of AI-based clinical tools will play a crucial role in paving the road to precision medicine in IBDs.

## 1. Introduction

Inflammatory bowel diseases (IBDs) are chronic disorders characterized by the mucosal inflammation of any segment of the gastrointestinal tract, leading to severe symptoms that could impair the patient’s quality of life. These are classified into two entities—Crohn’s disease (CD) and ulcerative colitis (UC)—which differ in clinical, endoscopic, and histopathological aspects, genetic and epidemiological features, and disease course [1,2]. The management of IBDs has evolved extensively in recent decades with the introduction of novel biologic and molecular therapies [3]. However, within the IBD management evolution pathway, the treatment objectives have changed from clinical remission and endoscopic mucosal healing to histologic remission [4,5]. In this context, endoscopy represents a benchmark for IBD diagnosis and disease monitoring, taking advantage of recent and important technological advancements.

Artificial intelligence (AI) has recently been extensively applied in endoscopy in the context of research settings, with the purpose of helping in the identification and characterization of colorectal polyps, increasing the rate of adenoma detection, and properly managing polyps from the perspective of clinical impact and costs [6]. AI is an umbrella term that includes several model types: natural language processing (NLP) data extraction from unstructured raw text with the generation of human language; the machine learning (ML) model, which enables the AI system to learn and improve from provided data and experiences automatically; the deep learning (DL) model, an ML application which trains the AI system through complex algorithms and deep neural networks, enabling the detection of complex patterns; artificial neural networks (ANNs) for image recognition and diagnosis; and convolutional neural networks (CNNs) for the automatic learning of complex patterns from raw images [7].

To date, the availability of AI systems for applications in endoscopy in the IBD setting has been limited [8]. The importance of a precise and reproducible assessment of mucosal healing to determine the activity of IBD and its response to therapy, thus guiding the choice of patient-focused treatment, has provided a research boost to the field of AI applications in the IBD setting. However, the available studies are characterized by heterogeneity in patient characteristics, study design, research methodology, AI systems, and endoscopic techniques, leading to gaps in the road from the research field to implementation in clinical practice and open debates on the actual usefulness of AI in IBD endoscopy.

In this narrative review, therefore, we assess recent advances in the application of AI-based integrated systems to assess and monitor patients with IBD during endoscopy.

## 2. Materials and Methods

A non-systematic review of the literature relating to the use of AI in endoscopy in the context of IBD, CD, and UC was undertaken. The PubMed and Scopus databases were searched using a combination of keywords such as ulcerative colitis, Crohn’s disease, inflammatory bowel disease, endoscopy, colonoscopy, capsule endoscopy, device-assisted enteroscopy, artificial intelligence, computer-aided detection, deep learning, machine learning, and neural networks. Studies that were published up until 31 December 2024, had their full text, and were in the English language were considered for this narrative review; these were categorized into the four main key applications of AI in the IBD endoscopy setting: (a) the diagnosis of IBD and the differential diagnosis between CD and UC and between IBD and other non-IBD colitis; (b) the assessment of endoscopic IBD severity; (c) the prediction of IBD histologic activity and clinical outcomes; and (d) the monitoring of disease and the detection of dysplasia occurring in IBD.

## 3. Limitations of Endoscopy and Advantages of AI in IBD

Endoscopy, encompassing colonoscopy—including magnification, image-enhanced, and microscopic advanced endoscopic techniques—video capsule endoscopy (CE), and device-assisted enteroscopy (DAE), is crucial for diagnosis, clinical management, treatment guidance, and disease monitoring in patients with IBD [9]. The availability of novel biological and molecular molecule-based therapies in the most recent decade has led to the improvement in the quality of life of patients with IBD [3]. At present, endoscopic and histologic remission is considered the primary outcome in the IBD research setting and clinical practice for the assessment of therapeutic efficacy (“treat-to-target” concept), besides clinical remission [10]. However, a significant discrepancy between endoscopic and histological remission has been observed using endoscopy with traditional equipment, as well as a high variability in the treatment objectives between endoscopists [11].

Due to this gap, which has a significant clinical impact on IBD management, recent improvements in endoscopic techniques and image-enhanced programs have also been advanced in the IBD setting.

High-definition (HD) endoscopy is currently recommended to assess IBD activity [12], but some concerns have arisen regarding its ability to accurately predict and determine the latter [13,14,15]. Using white-light endoscopy (WLE), histologically active disease was found in 21.6–23.1% of patients with UC in endoscopic remission [16,17]. In a meta-analysis, WLE did not significantly improve the pooled correlation coefficients between the endoscopic and histologic scores [18]. Chromoendoscopy, which enhances the mucosal superficial patterns and vascular networks using various dyes (dye-based chromoendoscopy, DCE) or electronic optical and digital color-filtering programs (virtual chromoendoscopy, VCE) [19], is currently widely available. However, in IBD endoscopy, contrast dyes present several disadvantages, such as (a) staining of non-inflamed tissue more than inflamed tissue by absorptive dyes (indigo carmine); (b) providing uneven mucosal surface coloring; (c) being time-consuming; (d) requiring endoscopists to have a high proficiency; and (e) lacking enhancement of the subepithelial capillary network [19,20,21]. New-generation endoscopes are usually implemented using VCE techniques (narrow-band imaging, NBI; Olympus Medical Systems, Tokyo, Japan; i-scan optical enhancement, OE; Pentax Medical, Tokyo, Japan; blue-light imaging and linked color imaging, BLI/LCI; Fujifilm, Tokyo, Japan) [22], and different VCE-based endoscopic scores have been proposed for the assessment of IBD activity, with a meta-analysis showing a higher accuracy in predicting histologic remission than WLE [18]. Foremost among them is the unique validated “Paddington International virtual ChromoendoScopy Score” (PICaSSO), evaluating the inflammatory-related vascular and mucosal changes in patients with UC [23]. This endoscopic score could be accurately reproduced with NBI and LCI/BLI, showing a good correlation coefficient with five histologic scores (Pearson’s correlation range: 0.77–0.79), and is therefore currently applied to all electronic VCE platforms [24,25]. However, considerable inter-observer variability, leading to the misevaluation of IBD endoscopic activity, limits a standardized endoscopic assessment using VCE.

Other techniques, such as probe-based confocal laser endomicroscopy (pCLE) and endocytoscopy (EC), show promising applications in the IBD setting. pCLE could be a useful tool for the real-time assessment of histologic activity in both UC and CD (“optical biopsy”), enabling differential diagnosis between UC and CD by visualizing the extent of inflammation and the morphology and density of crypts. The assessment of IBD activity using pCLE to visualize inflammation-related characteristics (such as the disruption of crypts with irregular and wider lumens and microvascular alterations) correlates well with histologic findings [26,27,28,29], even after medical treatment [30,31]. Similarly, through the use of an ultra-magnification endoscopic system in direct contact with the target lesion, EC can provide a highly accurate, real-time, in vivo pathological prediction [32]. The EC system score (ECSS), including characteristics linked to vessels and crypts, strongly correlates with histologic activity [33,34]. Moreover, pCLE and EC could enable the assessment of intestinal barrier permeability and the characterization of the inflammatory infiltrate, respectively [35,36]. However, both techniques require extensive diagnostic training for endoscopists, additional costs and time, and the use of intravenous fluorescein injection for pCLE or the application of mucolytic and contrast agents in EC.

In the context of CD, the topical application of fluorescently labeled adalimumab and vedolizumab during endoscopy allows the detection of membrane-bound TNF1 immune cells and α4β7 integrin, respectively, allowing for the prediction of therapeutic response [37,38]. However, although molecular endoscopic imaging (MEI) is under investigation, significant issues include the additional costs and procedural challenges in endoscopic examination.

In these contexts, the integration of AI systems in endoscopy could help IBD endoscopists because of their ability to analyze a large number of endoscopic images in real time, thus increasing the endoscopic diagnostic accuracy, providing an instantly available endoscopist-independent assessment of the mucosal disease activity, decreasing the inter-observer variability, assisting real-time histological evaluation, and reducing the reading time of CE videos. This would lead to the acquisition of more accurate data, better prediction of histologic remission and clinical outcomes, and improved insights for clinical and treatment decision making in IBD [39,40].

## 4. AI in Endoscopy for IBD and Differential Diagnosis

Evaluating endoscopic features to distinguish IBD from non-IBD colitis—particularly intestinal tuberculosis, which poses a diagnostic challenge in resource-limited settings—and differentiating CD from UC is complex, requiring precise interpretation by experienced clinicians, as well as intra- and inter-observer coherence.

Several studies, albeit retrospective, have been performed on the AI-based re-analysis of real-world endoscopic images to determine AI’s role in IBD diagnosis.

Different deep learning CNN models (Inception V3—Google AI, Mountain View, CA, USA; ResNet 50—Microsoft Research Asia, Beijing, China; VGG 19—Visual Geometry Group, Oxford, UK, and DenseNet 121—Cornell University, Ithaca, NY, USA) have been compared to determine the best prediction model to accurately distinguish UC from non-UC pathologies and inform the Mayo endoscopic score (MES) of disease severity (inactive/mild and moderate/severe), analyzing 8000 labeled endoscopic images from the HyperKvasir database (the largest available multi-class dataset of images and videos from the gastrointestinal tract—Bærum Hospital, Gjettum, Norway). The DenseNet 121 CNN model provided an area under the receiver operating curve (AUROC) of >0.99 and an accuracy of 98.3%. The addition of Gradient-Weighted Class Activation Maps (Grad-CAMs) improved the visual interpretation of the model over heatmaps [41]. In another similar study, evaluating 6000 endoscopic images from the KVASIR benchmark image dataset, the ResNet-50 CNN model achieved a differential diagnosis accuracy of 99.5% for UC on the validation set [42]. Guimarães et al. found no significant improvement in diagnostic accuracy for distinguishing between IBD and non-IBD colitis using CNN compared to endoscopists (70.9% vs. 72.1%). Only after implementation of the ML Gradient-Boosted Decision Tree (GBDT) approach based on five clinical parameters did the diagnostic accuracy significantly improve (76.6%; AUC = 0.838) [43]. Similarly, by implementing the CNN algorithm with the image pre-processing Pytorch framework (Meta AI, Astor Place, NY, USA) and visualizing the DL model through Grad-CAM (using 6617 colonoscopy images), the diagnostic accuracy for differentiating between CD from intestinal Behcet’s disease and tuberculosis was 65.15% for all images and 72.01% for typical images (*p* = 0.024) [44]. Inexperienced endoscopists could benefit from CNN-based ML in classifying CD and intestinal tuberculosis, as it showed a sensitivity and specificity of 90% and 77%, respectively [45]. The high yield of AI in distinguishing CD from intestinal tuberculosis has also been found in other studies, with a diagnostic accuracy ranging from 70% to 88.2% [46,47]. A novel classification and regression tree (CART) algorithm, incorporating laboratory, imaging, and endoscopic parameters, found that positive interferon-gamma release assays and circular ulcers are suggestive of intestinal tuberculosis, while involvement of ≥4 segments, along with longitudinal and aphthous ulcers, suggests CD. The overall differential diagnostic accuracy rate for distinguishing CD from intestinal tuberculosis was 88.6%. However, this model was trained on a small sample of patients [48].

AI applied in endoscopy also provides high accuracy in differentiating between CD and UC [7]. By training ResNet50 and ResNeXt-101, two different deep CNNs, on 29,414 and 57,330 colonoscopy still images, respectively, obtained from patients with CD and UC and healthy subjects, algorithms were developed to accurately differentiate these entities. The AI models demonstrated higher diagnostic performance than even the most competent endoscopists. The diagnostic accuracy for IBD ranged from 92% to 99.1% (vs. 92.2% for competent endoscopists and 78% for trainee endoscopists) per patient and from 90.4% to 90.9% (vs. 69.9% for competent endoscopists and 59.7% for trainee endoscopists) per image [49,50]. Importantly, the AI-based algorithm improved the diagnostic yield of non-expert endoscopists by 30.7% (per image) [49]. The accuracy in differentiating CD, UC, and healthy subjects was 92.39%, 93.35%, and 98.35%, respectively, compared to 91.70%, 92.39%, and 97.26% for the best-performing clinicians [49,50]. Another large retrospective DL-based (ResNet34/50/101) study of 11,404 IBD images achieved an accuracy of 90.6% for the differential diagnosis between UC and CD on the validation set. The SI CURA (“Soluzioni Innovative per la gestione del paziente e il follow-up terapeutico della Colite UlceRosA”) database was used as the gold-standard comparator [51]. A CAD method trained to specifically analyze the mucosal architecture on pCLE images from 23 patients with CD and 27 patients with UC, along with nine controls, achieved a sensitivity and specificity of 100% (95% CI = 93 to 100 and 95% CI = 66 to 100, respectively) for diagnosing IBD (*p* < 0.05 versus controls), as well as a 92% sensitivity (95% CI = 75 to 99) and a 91% specificity (95% CI = 72 to 99) for discriminating between patients with UC and those with CD [52].

Several studies have developed AI-based algorithms for IBD diagnosis using small-bowel and colonic CE videos with varying numbers of training images, comparing the results of endoscopists, both experts and fellows. Among them, one study evaluated the role of AI in video CE for UC, and only three studies were prospective. In the only prospective study including UC lesions (483,644 training datasets and 255,377 validating independent datasets from 31 video CE in 22 patients), the use of the DL ResNet50 framework, with a computational performance of 25 frames/s, achieved diagnostic accuracy rates of 99.2% and 98.3% for the training and validation datasets, respectively [53]. This DL model has been proven to be a useful tool for reducing the burden of image interpretation for endoscopists. The other two prospective studies, which included CD lesions, used the DL ResNet50 and AXARO (Augmented Endoscopy, Paris, France) frameworks, with 7744 training images and 470 images per patient from 130 patients, respectively. Applying the ResNet50 framework with a patient-dependent split of images for training, validation, and testing, the diagnostic sensitivity, specificity, and accuracy for CD-related ulcers were 95.7% (CI = 93.4–97.4), 99.8% (CI = 99.2–100), and 98.4% (CI = 97.6–99), respectively, with two expert readers as comparators. In this study, the diagnostic accuracy was equally high for both the small bowel and the colon [54]. The AXARO framework, applied in a prospective multi-center study of patients with suspected CD, achieved a 97.1% reduction in analyzable images and up to a 94% reduction in the reading time (pooled median review time = 3.2 min per patient) compared to fully read capsules. It also demonstrated a sensitivity and specificity of 92–96% and 90–93%, respectively, and an AUC of 0.91–0.94, highlighting its potential as a rapid tool for ruling out IBD in patients undergoing pan-enteric video CE [55]. The reported diagnostic sensitivity, specificity, and accuracy of CD-related lesions from the other retrospective studies assessing different CNN and DL models on video CE images varied from 88.2% to 98%, 89% to 99.9%, and 90.5% to 99%, respectively [56,57,58,59,60,61,62,63,64,65].

A summary of the studies and results on AI-based diagnosis and differential diagnosis in IBD endoscopy is reported in Table 1.

## 5. AI in Endoscopy for Assessment of IBD Endoscopic Activity

AI systems in IBD endoscopy have the potential to provide objective and reproducible grading of endoscopic activity in patients with IBD, particularly in the UC setting. Existing endoscopic scores for UC objectively grade the disease severity based on the presence of endoscopic findings without reflecting the picture of clinical severity within each endoscopic category. The most commonly used disease activity index for evaluating response to treatment is the MES, which is easy to apply but has the following notable drawbacks: lack of rigorous validation (poor inter- and intra-observer reliability), limited insertion length, inconsistent distinction between mild (MES 1) and moderate (MES 2) friability, and inability to distinguish between superficial and deep ulcers, reflecting only the most severely affected bowel segment [66,67]. Additionally, the Ulcerative Colitis Endoscopic Index of Severity (UCEIS) score suffers from wide inter-observer variability.

Several AI systems (CNN, deep NN, CAD, DL, support vector machine, residual network, class-based high-resolution network, long short-term memory, and visual geometry group) have been tested in the context of the different endoscopic scores for UC (MES, UCEIS, and PICaSSO), primarily using colonoscopy still images, with fewer studies using endoscopic videos [7]. Expert endoscopists or centrally read videos from clinical trials have been used as comparators. However, all but five of these studies were retrospective. The diagnostic accuracy and AUC of these AI models ranged from 86.54% to 94.5% and from 0.94 to 0.98, respectively [41,68,69,70,71,72].

Iacucci et al. trained a CNN algorithm on 1090 WLE images and VCE videos from 283 patients with UC to grade endoscopic remission/activity and predict histological remission/activity against the grading (using UCEIS and PICaSSO) and agreement provided by experts. This computer model accurately detected endoscopic remission according to UCEIS and PICaSSO, with a sensitivity of 72% and 79%, a specificity of 87% and 95%, and an AUROC of 0.85 and 0.94, respectively. The prediction of histologic remission was similar for the two scoring systems (80% and 85%), while the prediction of the risk of flare was similar to that based on the endoscopic scores provided by endoscopists [73]. However, this model was developed using videos recorded with the i-Scan platform (Pentax, Tokyo, Japan), whilst PICaSSO was recently reported to be valid for other VCE platforms [25]. An accurate distinction between UCEIS 0 (normal mucosa) and UCEIS ≥ 1 (active disease) and between UCEIS 0–3 (mild disease activity) and UCEIS ≥ 4 (moderate–severe disease activity) was achieved using an ML algorithm based on a multi-task learning framework, with accuracies of 90% and 98% (κ = 0.90 and κ = 0.96), respectively. The agreement for UCEIS subdomains (vascular pattern, bleeding, and erosion) was also high (κ ≥ 0.80) [74].

To express inflammation on a continuous scale (from 0 to 10) rather than as a categorical scale, thus providing a comprehensive UC inflammation assessment, a novel AI-based UC Endoscopic Gradation Scale (UCEGS) was generated to express UC severity by training a ranking-CNN using comparative information on UC severity from 13,826 pairs of endoscopic images. UCEGS correlates well with the MES 0–2 scores assigned by IBD expert endoscopists (Spearman’s correlation coefficient = 0.89) and shows a high correlation with the continuous values (0 to 10) provided by endoscopists. However, it offers less variability in estimates for mild- and moderate-disease images compared to the assessments made by endoscopists [75].

In other studies, AI-based differentiation between MES 0 (inactive disease) and MES 1–3 (active disease) achieved an accuracy of 94% (AUROC = 0.997), whereas the distinction between MES 0–1 (remission disease) and MES 2–3 (active disease) ranged from 83.7% to 93%, with an AUROC ranging from 0.966 to 0.998 [76,77,78]. Byrne et al. found that the best MES model performance was for severity levels 0 and 3, with specificities of 94.6% and 87.9% and sensitivities of 85.7% and 69.1%, respectively. For the best UCEIS model, performance was best at severity levels 0 and 5, with specificities of 93.9% and 79.1% and sensitivities of 88.2% and 58.6%, respectively. The accuracy for binary DL-based classification was 94% for MES 0–1 vs. MES 2–3 and UCEIS ≤ 3 versus UCEIS > 3 [79].

The high performance of a CNN-based CAD system in distinguishing MES 0 from MES 1–3 and MES 0–1 from MES 2–3 was confirmed in another study, with AUROCs of 0.86 (95% CI = 0.84–87) and 0.98 (95% CI = 0.97–98), respectively [80]. Interestingly, this performance was superior in the rectum compared to the right- and left-sided colon when distinguishing between MES 0 and MES 1–3 (AUROC = 0.92, 0.83, and 0.83, respectively). However, it was lower in the rectum than in the right- and left-sided colon when identifying MES 0–1 from MES 2–3 (AUROC = 0.99, 0.99, and 0.94, respectively). This could be attributed to topical treatment-induced modifications leading the inflamed mucosa to appear “patchy” or with “skip lesions”, making it more difficult to grade MES using CNN in the rectum accurately. The CNN performance was lower in patients receiving topical treatment compared to those who did not (AUROC = 0.89 and 0.96, respectively) [80].

An 89.1% (sensitivity = 82.3%; specificity = 92.2%) accurate differentiation between mucosal healing (MES 0) and MES 1 was achieved by combining DL- and ML-based CAD diagnostic systems, compared to the 83.3% accuracy achieved by trainee endoscopists [81]. This has prognostic importance, as a higher risk of disease relapse was recently observed in patients with MES 1 compared to those with MES 0, despite the fact that mucosal healing is defined as achieving either MES 0 or 1 [82]. The individual discrimination of MES 1, MES 2, and MES 3 in patients with UC was also achieved using a DL-based algorithm, with AUC values of 0.89, 0.86, and 0.96, respectively, and an overall accuracy of 77.2% [83]. Similarly, another DL-based algorithm, developed using 1672 raw videos from 124 patients with UC, predicted the Mayo Clinic Endoscopic Subscore (MCES) with a high degree of accuracy (AUROC = 0.84 for MCES ≥ 1, 0.85 for MCES ≥ 2, and 0.85 for MCES ≥ 3) [84].

The prediction of MES and UCEIS scores was also performed on full-length endoscopy videos prospectively collected from 249 patients with moderate-to-severe UC within a multi-center clinical phase 2 trial of mirikizumab. This was achieved through training a recurrent neural network (RNN) on score features. The RNN-assisted analysis generated a final endoscopic severity score, achieving high inter-rater agreement with human central readers and demonstrating excellent endoscopic accuracy in predicting endoscopic healing. Specifically, it showed a prediction accuracy of 97% for UCEIS and 95.5% for MES in distinguishing MES 0 from all other score levels [85].

However, scoring selected endoscopic images cannot fully reflect the distribution of inflammation across the entire intestine. Thus, Fan et al. developed a novel DL-based automatic scoring system for assessing inflammatory severity across 85 predetermined areas of different colon tracts from each video-based AI analysis. This system showed high accuracy in predicting each bowel segment’s score, with an accuracy of 86.54% for the MES-scored task and up to 90.7% for the UCEIS-scored task. Additionally, it visualized the distribution of intestinal inflammatory activity using a two-dimensional colorized image [86]. Furthermore, since UC endoscopic assessments report only the maximum severity observed, without taking into account the different extents and gradations of disease severity along the entire colon, Stidham et al. performed a post hoc computed vision analysis that spatially mapped the MES on endoscopic videos from the recent UNIFI trial. This trial evaluated the effects of ustekinumab as an induction and maintenance therapy in moderate-to-severe active UC. The analysis generated a cumulative disease score (CDS) that better quantified the mucosal injury and revealed significant correlations with MES. In addition, it proved more accurate in detecting changes following therapy compared to MES due to its ability to capture variations in the cumulative endoscopic disease severity within each MES level, thus requiring 50% fewer participants to estimate an endoscopic improvement between the ustekinumab and placebo arms. Stratification by pretreatment CDS predicted a greater effectiveness of ustekinumab over the placebo (*p* < 0.0001), with a more pronounced effect in severe disease compared to mild disease (*p* < 0.0001) [87]. Another AI-based scoring system, the Ulcerative Colitis Severity Classification and Localized Extent (UC-SCALE), was recently developed by Gutierrez Becker et al. [88] using 4326 sigmoidoscopy WLE videos from phase III Etrolizumab clinical trials. The UC-SCALE, which uses a quality filter for selecting readable images, a scoring system for assigning MCES to each frame, and a camera localization algorithm, achieved similar inter-rater agreement between the UC-SCALE and central and local experienced readers (κ ≥ 0.80). The strengths of this AI-based algorithm include its topological representation as a marker of disease severity and the moderate-to-high correlation of the Aggregated Disease Severity Score (ADSS), calculated using UC-SCALE, with several metrics. These include fecal calprotectin (rs = 0.50), C-reactive protein (rs = 0.45), patient-reported outcomes (rs = 0.45 for stool frequency and rs = 0.40 for rectal bleeding), physician global assessment (rs = 0.45), and total Geboes score (GS) (rs = 0.55) (*p* < 0.0001 for all metrics) [88].

Recently, colonic tissue oxygen saturation (StO2) was proposed as a measurement for endoscopic healing using the hypoxia imaging algorithm (EP-0002; Fujifilm, Tokyo, Japan), trained on 490 images from 100 patients with UC, based on the characteristic hypoxic microenvironment of the inflamed mucosa [89]. Rectal StO2, assessed by hypoxia imaging colonoscopy, significantly correlated with UC activity as evaluated by the Simple Clinical Colitis Activity Index (*p* < 0.001), as well as with its subscore, reflecting the urgency of defecation (*p* < 0.001), at a cut-off of 40.5% for both (AUROC = 0.72 and 95% CI = 0.61–0.84, and 0.74 and 95% CI = 0.62–0.87, respectively). Moreover, StO2 showed moderate accuracy in predicting both endoscopic and histologic activity, with an AUROC of 0.79 (95% CI = 0.74–0.84) for MES ≥ 2 and 0.76 (95% CI = 0.71–0.80) for UCEIS ≥ 2 at cut-offs of 45.5% and 47.5%, respectively. For GS ≥ 3, the AUROC was 0.72 (95% CI = 0.66–0.77) at s 45.5% cut-off. There was an inverse relationship between the StO2 values and MES/UCEIS and GS. However, the higher StO2 values recorded on the right side of the colon might have been influenced by the high concentration of bile components, which affected the detection of the spectral difference between oxyhemoglobin and deoxyhemoglobin for StO2 calculation.

In the CD setting, all studies employed a retrospective design. A multi-brand CNN-based algorithm, trained on 6772 images from single- or double-balloon DAE, was able to automatically detect relevant CD lesions, such as ulcers and erosions, with an accuracy of 98.7% and an AUC–precision recall curve of 1. The reported reading time was 293.6 frames per second, making this AI system potentially applicable in real-life DAE settings [90]. Compared to endoscopists, a DL model (EfficientNet-b528 −Google, Mountain View, CA, USA) combined with Grad-CAM architecture, trained on 155 small-bowel DBE still images from 628 patients with CD, achieved high accuracy in detecting ulcers (96.3%; 95% CI = 95.7–96.7%), non-inflammatory stenosis (95.7%; 95% CI = 95.1–96.2%), and inflammatory stenosis (96.7%; 95% CI = 96.2–97.2%). CD ulcers were also graded on a scale from 1 to 3 according to the ulcerated surface, size, and depth of the ulcers, achieving average accuracies of 87.3% (95% CI = 84.6–89.6%), 87.8% (95% CI = 85.0–90.2%), and 85.2% (95% CI = 83.2–87.0%), respectively [91]. Another study applied a combined DL-CNN and long short-term memory system to pCLE images (testing dataset of 780 images with inflammation and 344 control images) and successfully distinguished between normal and inflamed ileocolonic mucosa in patients with CD. This system showed potential for identifying mucosal healing in inactive CD, with a test accuracy of 95.3% and an AUROC of 0.98, along with irregular crypts and tortuous and dilated blood vessels being indicative of inflamed mucosa [92].

A summary of the studies and results assessing AI-based endoscopic activity using IBD endoscopy is reported in Table 2.

## 6. AI in Endoscopy for Assessment of IBD Histologic Activity and Prediction of Clinical Outcomes

As stated in the Selecting Therapeutic Targets in Inflammatory Bowel Disease (STRIDE-II) initiative of the International Organization for the Study of Inflammatory Bowel Diseases (IOIBD), combined clinical remission and endoscopic healing are required in long-term treatments [10]. However, persistent histologic inflammation beyond endoscopic mucosal healing is associated with an increased risk of clinical recurrence and the onset of dysplasia in the long term, especially in UC [5,93,94]. A study by Bryant et al. reported a 24% rate of histologically persistent inflammation despite endoscopic remission in patients with long-standing UC [13]. Some studies have reported that conventional WLE does not reliably identify histologic inflammation [95]. Advanced imaging endoscopic techniques such as NBI, i-Scan, CLE, and EC have demonstrated high diagnostic yield in predicting histologic severity, but only when used by expert IBD endoscopists [96,97,98,99]. Moreover, the assessment of histologic inflammation is characterized by high inter-observer variability between pathologists. Thus, AI in IBD endoscopy has the potential to standardize the assessment of histologic disease activity and predict clinical outcomes.

Maeda et al. retrospectively evaluated a CAD system (EB-01; Cybernet Systems Co., Ltd., Tokyo, Japan) to predict persistent histologic inflammation in UC, using images (525 for validation, from 187 patients) obtained using EC (520-fold ultra-magnifying endoscopy) and biopsy samples from six colorectal segments of each patient [100]. The diagnostic accuracy was 91% (95% CI = 83–95%), with very high reproducibility (κ = 1). However, the inter-observer consistency of the histologic GS was not assessed, and a central IBD expert pathologist was not involved. A more sophisticated version of this CAD system, the EndoBRAIN-UC system (Cybernet Systems Corp., Tokyo, Japan), was subsequently adopted in real time during ultra-magnifying colonoscopies in 52 patients with UC. It performed similarly to MES 0 for diagnosing histological healing (defined as GS < 3.1), with a sensitivity of 74.2%, a specificity of 93.8%, and an accuracy of 77.5% (vs. 79.2%, 90.6%, and 81.2%, respectively, for MES 0). This CAD model was also able to identify GS < 3.1 more accurately in MES 1 lesions (*p* = 0.0169) [101].

These figures were confirmed in prospective studies. Beyond the reported high DNN-based diagnostic accuracy (4187 WLE still images from 875 patients with UC) for endoscopic healing according to UCEIS (90.1%, 95% CI = 89.2–90.9) compared to endoscopists (κ = 0.798, 95% CI = 0.780–0.814), Takenaka et al. demonstrated its ability to identify histologic remission with an accuracy of 92.9% (95% CI = 92.1–93.7) and a κ coefficient of 0.859 (95% CI = 0.841–0.875) compared to the biopsy results [102]. In addition, this DNN model (known as deep neural ulcerative colitis, DNUC) predicted the patients’ prognosis. Patients with DNUC-based mucosal activity were at significantly higher risk of a worse prognosis (*p* < 0.001 vs. patients with mucosal healing), with hazard ratios for the risk of hospitalization, colectomy, steroid use, and clinical relapse (defined as partial MES ≥ 3, C-reactive protein ≥ 3 mg/L, and fecal calprotectin ≥ 250 mg/g) of 48.4, 46.4, 10.2, and 8.8, respectively, which were similar to those determined by expert endoscopists [103]. Subsequently, the same group applied DNUC to full video colonoscopies, confirming its ability to determine UCEIS, compared to centrally evaluated scoring by IBD expert endoscopists (intra-class correlation coefficient of 0.927; 95% CI = 0.915–0.938). Additionally, it accurately predicted histological remission in 81% of cases, with a sensitivity of 97.9% (95% CI = 97–98.5) and a specificity of 94.6% (95% CI = 91.1–96.9). Of note, the discrepancies between DNUC-based and central reader-based UCEIS scores could be attributed to the presence of inflammatory polyps and inadequate bowel preparation [104]. However, the DNN model was trained only to evaluate the presence or absence of histological inflammation, meaning a detailed histological assessment could not be conducted without biopsy specimens.

A good correlation between the AI-based endoscopic scores and histological activity was observed using a CAD-based algorithm that integrated pixel color data from the redness color map along with vascular pattern detection on WL images (Pentax Medical, HOYA Corporation, Tokyo, Japan). The outputted red density (RD) score correlated with the Robarts Histological Index (r = 0.74, *p* < 0.0001), MES (r = 0.76, *p* < 0.0001), and UCEIS (r = 0.74, *p* < 0.0001) [105]. In contrast to other CAD systems that require thousands of images, this RD approach needs less data, as the algorithm can be modulated sequentially during its development. The RD score also showed potential as an independent predictor of disease course during a follow-up period of five years [106]. An RD score cut-off ≥65 indicated a non-significant increase in the composite endpoint of treatment failure, which included mortality, colectomy due to refractory disease, disease flares, hospitalization, and change in treatment (HR = 2.0, 95% CI = 0.8–5.3). However, this endpoint was assessed retrospectively in only 39 patients with UC, and the results of the ongoing PROCEED-UC trial are awaited to confirm the accuracy and predictive value of the RD score in UC. A limitation of RD technology is its inapplicability to moving images and patients with CD due to the irregular distribution of inflammation and the non-dominant scoring system for endoscopic activity assessment in CD.

As known, the extent of changes in the mucosal peri-cryptal vasculature correlates with the degree of inflammation [52]. Single-wavelength endoscopy (SWE) performed using the prototype system EC-760R endoscope and the VP-7000 processor with a BL-7000 light source (Fujifilm, Tokyo, Japan) provided a real-time in vivo investigation of superficial mucosal crypts, peri-cryptal capillaries, and instances of bleeding (depth up to 5–200 mm). A novel CAD model based on non-magnifying SWE imaging, trained on the corresponding non-magnified HD-WLE images (6926 sets, from 112 UC patients), performed better than a CAD model based on WLE imaging for the assessment of histological remission (GS ≤ 2B.0), with a diagnostic accuracy of 83.3% at initial training (vs. 67.5% for CAD-WLE, *p* < 0.005) and 95.2% for the validation set [107]. Using the same endoscopic technology, Bossuyt et al. obtained a CAD-based diagnostic accuracy for histologic remission of 86%, compared to 74% and 79% using MES and UCEIS, respectively. Moreover, this CAD-based algorithm reached a 0.694 kappa statistic for correlation with the histologic GS, compared to the correlations between MES or UCEIS and GS (κ = 0.514 and 0.586, respectively) [108]. These studies demonstrate that AI systems could potentially support reducing the number of required biopsy samples and enable immediate therapeutic intervention.

Maeda et al. prospectively applied an AI system (EB-03 prototype; Cybernet Systems, Tokyo, Japan) in real time during colonoscopies in 134 patients with UC in clinical remission who were followed up for 12 months to directly predict clinical relapse (defined as partial MES > 2). The patients were categorized into AI-identified active and healing groups (74 and 61 patients, respectively). The clinical relapse rate was significantly higher in the AI-identified active group (28.4% vs. 4.9%; *p* < 0.001). The prediction of clinical relapse within 12 months was not significantly different between AI and histology following the analysis of biopsy specimens from 802 segments (accuracy of 58.5% vs. 65.2%; *p* = 0.316). The prediction of persistent histologic inflammation based on AI had a high accuracy of 93.8% [109]. The same group has more recently proposed an alternative real-time AI-based binary classification, which was applied during colonoscopies in 104 patients with UC in clinical remission: the AI-based vascular healing group and the AI-based vascular active group. Clinical relapse was significantly more frequent in the AI-based vascular active group (23.9% vs. 3%; *p* = 0.01). In patients with MES ≤ 1, the combination of endoscopic remission and vascular healing parameters provided the highest AUROC for predicting clinical relapse, compared to endoscopic remission alone or combined endoscopic and histologic remission (0.70 vs. 0.65 vs. 0.59) [110]. However, we must not forget that the therapeutic interventions that occurred in the follow-up period might have influenced these promising results. A fully automated three-class MES output (0, 1, and 2 or 3) during colonoscopies in 110 patients with UC in clinical remission, using the EB-UC2 AI prototype (Cybernet Systems, Tokyo, Japan) integrated with a 16-layer Visual Geometry Group network as the architectural framework, could stratify the risk of clinical relapse (defined as partial MES > 2) during the 12-month follow-up. The clinical relapse rates in patient groups classified as MES 0 and 1 were 3.2% (95% CI = 0.1–16.7%) and 24.5% (95% CI = 13.3–38.9%) (*p* < 0.01), whilst they were 16.2% (95% CI = 8.9–26.2%) and 50% (95% CI = 27.2–72.8%) in patients classified as MES 0 or 1 and MES 2 or 3 (*p* = 0.03). Furthermore, the inter- and intra-observer reproducibility of non-IBD endoscopists was improved (correlation coefficients = 0.84–0.86 with AI vs. 0.64–0.76 without AI, and 0.89 with AI vs. 0.76 without AI, respectively) [111].

A summary of the studies and results on AI-based histologic activity assessment and prediction of clinical outcomes in IBD endoscopy is reported in Table 3.

## 7. AI in Endoscopy for IBD Surveillance and Assessment of Dysplasia

Endoscopic surveillance in patients with IBD should be mandatory due to the increased risk of developing colorectal cancer (CRC) [112,113], with an exponential trend rate according to IBD duration (1%, 4%, and 14% at 10, 20, and 30 years, respectively, from IBD diagnosis) [94]. Moreover, IBD-associated dysplasia is often difficult to detect and grade due to chronic inflammation, flat morphology, and margins that are not clearly distinguishable from the surrounding mucosa [114]. Regarding the recent implementation of AI systems into endoscopy, leading to improvement in the detection of colorectal lesions, AI may play a role in detecting early-stage IBD-related dysplasia, identifying patients with IBD who should undergo surveillance colonoscopy, and developing appropriate strategies for surveillance. However, the current data are from case reports and small studies only.

In two case reports, the EndoBRAIN and EndoBRAIN-EYE CAD systems (Cybernet Systems, Tokyo, Japan), previously used successfully for the detection and characterization of colorectal polyps, applied during EC (CF-H290ECI; Olympus, Tokyo, Japan) and high-definition endoscopy (CFHQ290ZI; Olympus, Tokyo, Japan) with NBI in two patients with a long-term history of pan-colitis UC successfully detected a colonic neoplastic lesion and a flat lesion with low-grade dysplasia [114,115]. Guerrero Vinsard et al. retrained an original CADe system (CSPDarkNet53, with cross-stage partial networks) for patient-specific IBD, testing it on HD-WLE images of colorectal lesions in non-IBD patients. The system was evaluated using 1266 HD-WLE and 426 DCE still images of histologically proven dysplastic colorectal lesions in the context of mild-to-moderate mucosal inflammation, achieving good performance with HD-WLE images, showing a 96.8% diagnostic accuracy and a 0.85 AUC (against 77.8% and 0.65, respectively, when using DCE images). Interestingly, the IBD-CADe architecture showed a higher sensitivity in detecting lesions ≤ 10 mm compared to those ≥10 mm (93% for ≤5 mm, 91% for 6–10 mm, and 85% for ≥10 mm). Of note, IBD lesions ≥ 10 mm are often pseudopolyps with a mixed morphology or stalks and overlying mucus. Furthermore, IBD-CADe performed better for lesion types Ip, Is, and IIa (Paris classification), whilst IIb or mixed-morphology lesions were more frequently missed. In addition, it was capable of detecting serrated lesions (epithelial changes and adenomas), even if with a lower true-positive rate (85.7%) than for other dysplastic and non-dysplastic lesions (≥90%). Most missed lesions had higher inflammation scores (missing rates of 7.3% for MES 0, 1.5% for MES 1, and 8.7% for MES 2 and 3) [116]. Another DL model (RetinaNet architecture with ResNet-101 backbone, trained on 478 images from 30 IBD patients) classified lesions into “neoplastic” and “non-neoplastic” with a 93.5% and 87.5% sensitivity and an 80.6% specificity for lesion detection and lesion characterization, respectively [117]. The prediction of neoplasia specifically occurring in IBD was also achieved through a deep CNN-based AI system (EfficientNet-B3), producing a binary classification into “adenocarcinoma or high-grade dysplasia” and “low-grade dysplasia or sporadic adenoma/normal mucosa.” Compared to the diagnostic accuracy provided by four experts and three non-expert endoscopists (for 186 test set images: 77.8%, 95% CI = 74.7–80.8, and 75.8%, 95% CI = 72–79.3, respectively), the diagnostic accuracy of the CNN-based dual-classification was higher, at 79% (95% CI = 72.5–84.6) [118]. However, although in this study the diagnosis of colorectal lesions was performed using p53 and Ki-67 immunostaining, a genetic background analysis was not performed; thus, sporadic colorectal neoplasia might have been included. Such AI systems, if further improved, could help endoscopists, mainly non-experts, in identifying colitis-associated dysplasia or CRC, avoiding unnecessary biopsies.

Finally, the integration of text-based electronic medical records (EMRs) with an NLP- based document-level classification, using the automated retrieval console (ARC) software (available as open source software at http://research.maveric.org/mig/arc.html (accessed on 27 December 2024)) allowed the differentiation between surveillance and non-surveillance colonoscopies, with a recall of 0.77 (95% CI = 0.66–0.85), a specificity of 0.88 (95% CI = 0.80, 0.93), and a precision of 0.80 (95% CI = 0.72, 0.90) for a total of 575 colonoscopy pathology reports in 195 IBD patients, compared to the manual review of pathology reports [119].

## 8. Other AI Applications in IBD Endoscopy

With the development of digital pathology, AI algorithms are increasingly employed in histopathological assessments on IBD biopsy specimens. Recently, the Paddington International virtual ChromoendoScopy ScOre (PICaSSO) Histologic Remission Index (PHRI), based only on the presence or absence of neutrophils’ infiltration in the lamina propria and epithelium, was developed using AI within a prospective multi-center study evaluating biopsy samples from 307 UC patients [23]. For each biopsy, from each rectum and sigmoid segment, the worst histologic features were scored using the GS, Robarts Histological Index (RHI), Nancy Histological Index (NHI), ECAP (extent, chronicity, activity and plus) score, and Villanacci Simplified Score. The PHRI score showed a high inter-rater agreement among pathologists (intra-class correlation coefficient of 0.84, 95% CI: 0.78 to 0.90) similarly to RHI and NHI, the strongest correlation with the endoscopic activity according to MES, UCEIS, and PICaSSO (*p* < 0.05), and the highest correlation with the long-term clinical outcomes (hospitalization, colectomy, and changes in medical therapy due to flare-up), as a PHRI of 1 could accurately stratify the risk of adverse outcomes up to a 12-month follow-up. Subsequently, the PHRI score’s determination through a novel CNN-based architecture DL model detecting neutrophils (training set of 138 biopsies) allowed the differentiation between active and quiescent UC, with 78% sensitivity, 91.7% specificity, and 86% accuracy. PHRI could be successfully implemented into AI models.

Subsequently, Iacucci et al. validated the PHRI on 375 digitalized biopsies using a CNN-VGG16 architecture. The AI classifier accurately distinguished mucosal remission from inflammation with an 89% (95% CI = 0.82–0.94) sensitivity and an 85% (95% CI = 0.80–0.89) specificity (compared to 94% and 76% for RHI and 89% and 79% for NHI, respectively), as well as predicted the corresponding endoscopic remission and activity with an AUROC of 79% (95% CI = 0.75–0.83) and 82% (95% CI = 0.78–0.86) for UCEIS and PICaSSO, respectively. Moreover, it predicted disease flare-ups (hospitalization, UC-related surgery, and changes in UC therapy) for up to 12 months, with a better hazard ratio according to AI-assessed PHRI for histologic remission and activity groups (*p* < 0.001) (4.64 vs. 3.56 according to pathologist-assessed PHRI) [120]. However, the limitations of this AI-based system include its inability to distinguish different UC severity grades and detect the presence of dysplasia, as well as the lack of worldwide availability of digital pathology. Another DL-based histologic score focused on the detection of eosinophils in sigmoid biopsy specimens (88 UC patients with histologically active disease according to GS and RHI) [121], achieving high agreement with pathologists’ eosinophil counts (interclass correlation coefficients = 0.805–0.917). The eosinophil density was not correlated with histologic activity or biologic use (infliximab, adalimumab, or vedolizumab) but with the disease extent (146.2 cells per mm^2^ for Montreal E3 vs. 88.2 cells per mm^2^ for Montreal E2; *p* = 0.005) and corticosteroid use (62.9 cells per mm^2^ vs. 124.1 cells per mm^2^ in non-corticosteroid use; *p* = 0.006). The DL-based quantification of goblet cell mucus area, as mucin depletion represents a histological risk factor for clinical relapse in MES 0–1, on whole slide images of biopsies (114 UC patients in clinical and endoscopic remission) was proposed by Ozaki et al. for the prediction of clinical relapse (defined as partial MES ≥ 3) [122]. The goblet cell ratio (goblet cell mucus area/epithelial cell and goblet cell mucus area) in specimens of the cecum, ascending colon, and rectum in relapsed patients was lower compared to relapse-free patients (*p* = 0.010, 0.027, and <0.01, respectively) [123].

Despite recent advances in treatment options, including small molecules and new biologic agents, the response rate to therapy remains modest, and a significant number of patients require a change in treatment over time. However, limited evidence is currently available to guide therapeutic choices. The Endo-Omics study (15 CD and 14 UC patients) demonstrated that in vivo CAD quantitative analysis of pCLE images, including abnormalities in vessel tortuosity, crypt morphology, and fluorescein leakage, predicted the response to anti-TNF or anti-integrin α4β7 therapy after 12 to 14 weeks, with an accuracy of 85% and 80% in patients with UC and CD, respectively (AUROC = 0.93 and 0.79). The ex vivo CAD analysis of fluorescein isothiocyanate-labeled infliximab and vedolizumab staining on the biopsy specimens showed that baseline increased binding of labeled biologics could predict the response to therapy, with a 77% accuracy only in patients with UC (AUROC = 83% vs. 58% in patients with CD) [124]. A spatiotemporal ML-based analysis of CE videos from 101 newly diagnosed and treatment-naïve patients with CD, followed up for six months using the TimeSformer computer vision algorithm (Facebook Research, Menlo Park, California, USA), achieved better prediction for the need of biological therapy compared to the Lewis score (human readers’ grading) and fecal calprotectin (AUROC = 0.86, 0.70, and 0.74, respectively) [125].

## 9. Conclusions and Future Directions

Overall, as outlined in this review, the implementation of AI algorithms in IBD endoscopy offers substantial benefits, which could revolutionize the management and precision of medicine in the context of IBD. These benefits include the following: (1) enhanced diagnostic accuracy, assisting in the detection of subtle mucosal lesions, ulcers, and inflammation which may be missed by human observers during examinations, differentiating between UC and CD and between IBD and non-IBD colitis, and helping non-IBD expert endoscopists; (2) real-time assessments with prompt decisions about management and therapy based on rapid on-site diagnostic outcomes; (3) standardized and objective evaluation of the disease activity, automatically determining scoring systems like MES or UCEIS or producing new AI-based scores, with a final reduction in the intra- and inter-observer variability across endoscopists (and pathologists); (4) improved efficiency, predicting histologic activity based on endoscopic findings, reducing the need for multiple biopsies, and decreasing the workload for endoscopists and pathologists; and (5) enhanced detection of flat and subtle pre-cancerous lesions, which are visually challenging [8]. Moreover, AI could significantly reduce the reader times of CE videos, with a diagnostic accuracy of up to 99.9%, although the risk of missing lesions should still be assessed. These AI-related strengths would also impact IBD clinical trials beyond clinical practice through helping in central reading [126].

In IBD management, the accurate assessment of mucosal inflammation and healing—both endoscopic and histologic—is crucial for guiding therapeutic and surveillance strategies [5,13,14,15,16,17,18]. However, despite the growing application of AI algorithms in digestive endoscopy, their added value in IBD endoscopy remains unclear, as endoscopic IBD-specific AI algorithms are lacking. Most of the evidence regarding AI applications in IBD endoscopy is provided by retrospective, low-quality, or small-sample studies, particularly for assessing the severity of IBD activity, which remains the most investigated endpoint in AI-supported IBD endoscopy [65,66,67,68,69,70,71,72,73,74,75,76,77,78,79,80,81,82,83,84,85,86,87,88,89,90,91,92]. In a recent meta-analysis, the AI diagnostic accuracy for mucosal healing in UC had high sensitivity and specificity but a low yield in accurately differentiating severity grades (e.g., grade 0 vs. 1 and grade 2 vs. 3). Moreover, the meta-analysis detected a moderate-to-high heterogeneity between studies [127]. Similarly, due to the availability of only a few prospective studies, the integration of AI algorithms into clinical practice for diagnosing IBD and distinguishing between IBD and non-IBD colitis or between UC and CD is still in its early stages [41,42,43,44,45,46,47,48,49,50,51,52,53,54,55,56,57,58,59,60,61,62,63,64]. The prediction of histological remission and response to therapy through HD or ultra-HD endoscopy or real-time histology using pCLE or EC technologies based on AI-based histologic scores also lacks substantial evidence in this preliminary stage [101,102,103,104,105,106,107,108,109,110,111]. The accuracy of AI-based detection and characterization of dysplasia in the IBD setting requires further improvements, as its use is limited by the difficulty in differentiating mucosal and microvascular changes caused by inflammation from those due to malignancy [128]. AI algorithms developed for detecting and characterizing colonic neoplasms may be unsuitable for dysplasia/neoplasia in IBD. Several other challenges also need to be addressed, including the quality of input and output images, which can be compromised by bowel preparation, and the variability in the training datasets on which AI performance depends, which affects the efficiency of AI algorithms in the IBD context. Ethical considerations also need to be considered when integrating AI into clinical and endoscopic practices, which require regulatory approval, data protection measures, and patient privacy. Moreover, elevated AI training and workflow changes, rigorous AI testing, approval by regulatory bodies, extensive external validations in real-world clinical settings, multidisciplinary approaches, and randomized studies and meta-analyses are required before deployment in clinical practice to determine whether AI can effectively improve diagnostic accuracy and forecast clinical outcomes during IBD endoscopy. However, the implementation of AI algorithms in IBD endoscopy holds considerable potential for advancing patient-tailored treatment, monitoring, and surveillance strategies, ultimately improving patient outcomes. This is particularly true with the use of multi-modal AI systems that integrate endoscopic imaging from HD and ultra-HD procedures, patient-level data, radiologic images, and genetic and omics data.

## Figures and Tables

**Table 1:** Studies on artificial intelligence-based diagnosis and differential diagnosis in inflammatory bowel disease endoscopy.

Study	Year of Publication	Study Design	Endoscopic Technique	Artificial Intelligence Platform	N. of pts	Study Endpoints	Results	Comparator
Sutton et al. [41]	2022	Retrospective Single-center	WLE	Inception-V3 ResNet-50 VGG-19 DenseNet-121	N/R	Diagnosing UC vs. non-UC	AUROCs: =0.999 DenseNet-121 =0.9978 Inception-V3 =0.9958 ResNet-50 =0.9988 VGG-19	One expert and two trainee endoscopists
Sharma et al. [42]	2023	Retrospective Single-center	WLE	ResNet-50 VGG-16 Inception-V3	N/R	Diagnosing UC, polyps, esophagitis, and healthy colons	Accuracies: Validation set: =99.84% ResNet-50 =92.18% VGG-16 =94.6% Inception-V3 Test set: =99.16% ResNet-50 =93.44% VGG-16 =96.82% Inception-V3	Kvasir database
Guimarães et al. [43]	2023	Retrospective Single-center	WLE	DenseNet + GBDT (five clinical parameters)	Training: 444 pts Test: 50 pts	Differentiating between IBD and infectious and ischemic colitis	Overall accuracy: =70.9% DenseNet =79.2% GBDT algorithm =76.6% DenseNet + GBDT	Three expert endoscopists
Kim et al. [44]	2021	Retrospective Single-center	WLE	ResNet-34	211 CD, 299 intestinal BD, and 217 ITB pts	Differentiating between CD and intestinal BD and ITB	AUROC = 0.78–0.86 Accuracies: All images: =65.15% CD vs. BD vs. ITB =78.15% CD vs. BD =78.09% BD vs. ITB =69.59% CD vs. ITB Typical images: =72.01% CD vs. BD vs. ITB =85.62% CD vs. BD =83.52% BD vs. ITB =75.66% CD vs. ITB	Two experienced endoscopists
Tong et al. [45]	2020	Retrospective Single-center	WLE	CNN using the Phyton framework	6399 pts	Differentiating between UC, CD, and ITB	AUROCs: =0.936 UC vs. CD =0.892 UC vs. ITB =0.910 CD vs. ITB	Endoscopists (number and expertise N/R)
Lu et al. [47]	2023	Retrospective Single-center	WLE	Text-CNN	875 CD 396 ITB	Differentiating between CD and ITB	Accuracies: =83% standard TextCNN (Robust) =70% noisy TextCNN (Robust)	Endoscopists (number and expertise N/R)
Lu et al. [48]	2021	Retrospective Single-center	WLE	CART model	Training: 84 CD, 84 ITB Validation: 22 CD, 22 ITB	Differentiating between CD and ITB	Accuracy = 88.64% ≥4 segments involved, longitudinal ulcers, aphthous ulcers suggestive of CD	Endoscopists (number and expertise N/R)
Ruan et al. [49]	2022	Retrospective Multi-center	WLE	ResNet-50	Training: 1358 pts Test: 218 pts External data: 196 pts	Differentiating between UC, CD, and normal colons	Accuracies: =99.1% per patient (vs. 78% and 92.2% of trainee and competent endoscopists) =90.4% per lesion (vs. 59.7% and 69.9% of trainee and competent endoscopists)	Five expert and five trainee endoscopists
Wang et al. [50]	2022	Retrospective Multi-center	WLE	ResNeXt-101	Training: 217 CD pts, 279 UC pts, and 100 healthy controls	Differentiating between CD, UC, and normal colons	Accuracies: =92.04% per image =90.91% per patient =92.39% CD per image =93.35% UC per image =98.35% normal per image (vs. 91.7% CD, 92.39% UC, and 97.26% normal for best-performing endoscopists)	Six endoscopists of different seniorities
Chierici et al. [51]	2022	Retrospective Multi-center	WLE	ResNet-18 ResNet-34 ResNet-50 ResNet-101 ResNet-152	N/R	Differentiating between CD, UC, and normal colons	Matthews correlation coefficient: >0.9 IBD vs. normal and UC vs. normal (ResNet34-50-101 best performing) >0.6 UC vs. CD (ResNet34-50-101 best performing)	Endoscopists (number and expertise N/R)
Quénéhervé et al. [52]	2019	Retrospective Single-center	CLE	CAD system	23 CD, 27 UC pts, and 9 healthy controls	Diagnosing IBD Differentiating between UC and CD	IBD diagnosis: Sensitivity = 100% Specificity = 100% CD vs. UC: Sensitivity = 92% Specificity = 91%	N/A
Higuchi et al. [53]	2022	Prospective Single-center	CE	ResNet-50	22 UC pts	Diagnosing UC	Accuracies: =99.2% training =98.3% validation	Five well-trained endoscopists
Majtner et al. [54]	2021	Prospective Multi-center	CE	ResNet-50	38 pts with suspected or known CD	Diagnosing CD	Accuracies: =98.58% random split =98.38% patient split Agreement on severity disease: κ = 0.90 random split κ = 0.72 patient split	Three experienced gastroenterologists
Brodersen et al. [55]	2023	Prospective Multi-center	CE	AXARO^®^ framework	131 suspected CD	Diagnosing IBD and CD	AUROCs: =0.91–0.94 CD =0.93–0.94 IBD Sensitivity: =92–96% CD =97% IBD Specificity: =90–83% CD =90–91% IBD	Two specialized observers
Charisis et al. [56]	2016	Retrospective Single-center	CE	Hybrid Adaptive Filtering- Differential Lacunarity analysis	13 CD pts	Diagnosing CD	Accuracy: =93.8% Precision: =92.6%	N/R
Aoki et al. [57]	2019	Retrospective Single-center	CE	CNN based on Single-Shot Multibox Detector	65 CD pts	Diagnosing CD	AUROC: =0.958 Accuracy: =90.8% at a cut-off value of 0.481 for the probability score	Two expert endoscopists
Klang et al. [58]	2020	Retrospective Single-center	CE	Xception CNN	49 CD pts	Diagnosing CD	AUROCs: =0.99 random split =0.94–0.99 patient level Accuracy: =95.4–96.7%	One experienced endoscopist
Barash et al. [59]	2021	Retrospective Single-center	CE	Deep Ordinal Ranking model	49 CD pts	Grading of ulcer severity	Agreement between consensus reading and automatic algorithm = 67%; AUROCs: =0.958 grade 1 vs. grade 3 ulcer severity =0.565 grade 1 vs. 2 ulcer severity =0.939 grade 2 vs. 3 ulcer severity	Two and three capsule readers (experiments 1 and 2)
Klang et al. [60]	2021	Retrospective Single-center	CE	EfficientNet-B5	N/R	Detecting CD strictures	AUROCs: =0.971 strictures vs. non-strictures =0.989 strictures vs. normal mucosa =0.942 strictures vs. all ulcers; AUROCs between different grades of ulcers: =0.992 for mild grade =0.975 for moderate grade =0.889 for severe grade	N/R
De Maissin et al. [61]	2021	Retrospective Single-center	CE	ResNet-34 VGGNet-16-19	63 CD pts	Diagnosing IBD vs. non-IBD	Overall precision = 93.7%; Overall k = 0.79; Accuracies: =94.58% ResNet-34 = 94.4% VGGNet-16 = 94.35% VGGNet-19	Three IBD experts
Ferreira et al. [62]	2022	Retrospective Multi-center	CE	CNN using Xception model	N/R	Detecting CD ulcers and erosions	Precision = 97.1% Accuracy = 92.4%, Detection of ulcers: Sensitivity = 83% Specificity = 98% Detection of erosions: Sensitivity = 91% Specificity = 93%	Three CE experts
Kratter et al. [63]	2022	Retrospective Single-center	CE	EfficientNet-B4	N/R	Detecting CD ulcers	Average AUROC = 0.99 Average mean patient accuracy = 97.4%	Gastroenterology fellows supervised by capsule experts (number N/R)
Ribeiro et al. [64]	2022	Retrospective Multi-center	CE	CNN using Xception model	124 CD pts	Detecting CD ulcers and erosions	Accuracy = 99.6% AUROC = 1.00	Three CE experts
Wang et al. [65]	2019	Retrospective Single-center	CE	Second glance detection framework	1504 pts (1076 ulcers, 428 normal mucosa)	Detecting CD ulcers	AUROC = 0.9469 (vs. 0.9014 Faster-RCNN and 0.8355 SSD-300) Accuracies: =90.1% overall =85% for ulcers <1% of the full image size =92% for ulcers >1% of the full image size	N/R

WLE: white-light endoscopy; N/R: not reported; UC: ulcerative colitis; AUROC: area under the receiver operating characteristic; GBDT: Gradient-Boosted Decision Tree; IBD: inflammatory bowel disease; CD: Crohn’s disease; BD: Behcet’s disease; ITB: intestinal tuberculosis; CNN: convolutional neural network; CART: classification and regression tree; CLE: confocal laser endomicroscopy; and CE: capsule endoscopy.

**Table 2:** Studies on artificial intelligence-based endoscopy for the assessment of endoscopic activity in inflammatory bowel disease.

Study	Year of Publication	Study Design	Endoscopic Technique	Artificial Intelligence Platform	N. of Patients	N. of Images	Study Endpoints	Results	Comparator
Sutton et al. [41]	2022	Retrospective Single-center	WLE	Inception-V3 ResNet-50 VGG-19 DenseNet-121	N/R	851 still images from the HyperKvasir dataset	Distinguishing MES 0–1 (inactive/mild) from 2 to 3 (moderate/severe) in UC	AUROCs: =0.90 DenseNet-121 =0.90 Inception-V3 =0.66 ResNet-50 =0.83 VGG-19	One expert and two trainee endoscopists
Higuchi et al. [53]	2022	Prospective Single-center	CE	ResNet-50	22 UC pts	Training: 483,644 images Validation: 255,377 images	Assessing endoscopic severity in UC along the entire length of the colon	Accuracy validation dataset: =99.4% MES 0 =94.8% MES 1 =91.3% MES 2 =95.2% MES 3	Five well-trained endoscopists
Barash et al. [59]	2021	Retrospective Single-center	CE	Deep Ordinal Ranking model	49 CD pts	7391 CD images; 10,249 normal mucosa images	Grading of ulcer severity in CD	Overall agreement between manual reading and automatic algorithm = 67% AUROCs: =0.958 grade 1 vs. grade 3 ulcer severity =0.565 grade 1 vs. 2 ulcer severity =0.939 grade 2 vs. 3 ulcer severity	Three capsule readers
Kim et al. [67]	2023	Retrospective Single-center	WLE	VGG-16	492 UC pts	984 still images	Differentiating MES 0 vs. 1	F1-score = 0.92 AUROC = 0.97 AUPRC = 0.98 External test: F1-score = 0.89 AUROC = 0.86 AUPRC = 0.97	Three IBD experts and seven fellow doctors External test: HyperKvasir dataset
Wang et al. [68]	2023	Retrospective Single-center	WLE	High-Resolution Network with Class-Balanced Loss	308 UC pts	12,163 still images	Assessing endoscopic activity in UC	MES 0 vs. 123: Accuracy = 93.73% κ = 0.8433 AUROC = 0.9754 MES 01 vs. 23: Accuracy = 95.1% κ = 0.8836 AUROC = 0.9834	Three IBD experts
Polat et al. [69]	2023	Retrospective Single-center	WLE	ResNet-18 ResNet-50 DenseNet-121 Inception-V3 MobileNet-V3-large	564 UC pts	11,276 still images	Assessing endoscopic activity in UC	QWK Mayo subscores = 0.847 (MobileNet-V3-large)—0.854 (ResNet-18) κ remission = 0.834 (MobileNet-V3-large)—0.852 (ResNet-50)	Two experienced gastroenterologists
Qi et al. [70]	2023	Retrospective Multi-center	HD endoscopy	ViT network	768 UC pts	15,120 still images	Predicting MES in UC	AUROCs: =0.998 MES 0 =0.984 MES 1 =0.973 MES 2 =0.990 MES 3 Overall accuracy = 87.1% (vs. 90.8% of endoscopists)	Six expert endoscopists
Turan et al. [71]	2022	Retrospective Single-center	HD endoscopy	UC-NfNet	N/R	673 still images from the HyperKvasir dataset	Classifying colonoscopic UC images	Accuracy = 84.91% Precision score = 85.27% Recall score = 84.91% F1-score = 85.14% MCC = 79.89%	Five board-certified endoscopists with <5 years of experience
Iacucci et al. [72]	2023	Retrospective Multi-center	WLE and VCE videos	ResNet-50	283 UC pts	1090 endoscopic videos (67,280 frames)	Distinguishing UC endoscopic remission (ER)	WLE videos: AUROC = 0.85 (UCEIS ≤ 1) Cohen’s κ coefficient = 0.51 VCE videos: AUROC = 0.94 (PICaSSO ≤ 3) Cohen’s κ coefficient = 0.73	Experienced endoscopists from the PICaSSO group
Patel et al. [73]	2022	Prospective Single-center	HD endoscopic videos	Multi-task learning algorithm (MLA)	73 UC pts	38,124 frames	Distinguishing UCEIS 0 vs. active disease, UCEIS 0–3 vs. moderate/severe disease	UCEIS 0 vs. ≥1: Accuracy = 0.90 κ = 0.90; UCEIS 0–3 vs. ≥4: Accuracy = 0.98 κ = 0.96; MLA vs. experts: Total UCEIS κ = 0.92 Vascular pattern κ = 0.81 Bleeding κ = 0.83 Ulceration κ = 0.88	Three IBD experts
Takabayashi et al. [74]	2024	Retrospective Multi-center	HD endoscopy	Ranking-CNN	812 UC pts	13,826 pairs of still images	Grading UC severity by UC Endoscopic Gradation Scale (UCEGS)	Spearman’s correlation coefficients: =0.89 UCEGS vs. MES =0.96–0.98 UCEGS vs. IBD expert endoscopists	Seven IBD expert endoscopists
Lo et al. [75]	2022	Retrospective Single-center	WLE	Inception Net-V3 EfficientNet-B0, B1, B2, B3, and B4	467 UC pts	1484 still images	Distinguishing active vs. healed mucosa; differentiating levels of endoscopic disease activity	Accuracies:nception Net-V3: =0.81 all MES =0.94 MES 0 vs. 1–3 =0.91 MES 0–1 vs. 2–3 EfficientNet-B: =0.82–0.86 all MES EfficientNet-B2: =0.84 all MES =0.94 MES 0 vs. 1–3 =0.93 MES 0–1 vs. 2–3	Two IBD experts
Yao et al. [76]	2021	Retrospective Multi-center	HD endoscopic videos	Inception-V3	157 UC pts	175 videos	Grading endoscopic UC disease	Informative image classifier: AUROC = 0.93; Correct prediction of MES: 78%; Correct classification MES 0–1 vs. 2–3: 83.7% Accuracies: =0.947 MES 0 =0.888 MES 1 =0.678 MES 2 =0.711 MES 3	Two IBD experts
Stidham et al. [77]	2019	Retrospective Single-center	HD endoscopy	Inception-V3	3082 UC pts	16,514 still images; 30 endoscopic videos	Grading endoscopic UC disease	MES 0–1 vs. MES 2–3: AUROC = 0.97 still images/0.966 videos Agreement CNN vs. experts: κ = 0.84 still images/0.75 videos	Two IBD experts
Byrne et al. [78]	2023	Prospective Single-center	HD endoscopy	EfficientNet-B3	N/R	134 videos (1,550,030 frames)	Predicting MES and UCEIS in UC pts	At section level: MES κ = 0.886 UCEIS κ = 0.904 Vascular pattern κ = 0.905 Bleeding κ = 0.754 Erosions and ulcers κ = 0.800; At video level: MES κ = 0.821 UCEIS κ = 0.646 Vascular pattern κ = 0.879 Bleeding κ = 0.391 Erosions and ulcers κ = 0.600	One global central reading expert, six gastrointestinal specialists, and twenty gastrointestinal trainees
Ozawa et al. [79]	2019	Retrospective Single-center	WLE	CNN-based CAD system on GoogLeNet architecture	841 UC pts	26,304 still images	Identifying normal mucosa (MES 0) vs. healing state (MES 0–1)	AUROCs MES 0 vs. 1–3: =0.86 overall =0.92 rectum =0.83 right side =0.83 left side =0.95 topical treatment =0.95 no topical treatment AUROCs MES 0–1 vs. 2–3: =0.98 overall =0.99 rectum =0.99 right side =0.94 left side =0.89 topical treatment =0.96 no topical treatment	N/R
Huang et al. [80]	2021	Retrospective Single-center	HD endoscopy	DNN, support vector machine, k-nearest neighbor network	54 UC pts	856 still images	Diagnosing mucosal healing in UC	Accuracies: =94.5% MES 0–1 vs. 2–3 =89.1% MES 0 vs. 1	Two reviewers
Bhambhvani et al. [82]	2021	Retrospective Single-center	HD endoscopy	ResNeXt-101	777 active UC pts	777 representative still images from the HyperKvasir dataset	Grading individual MES in UC	AUROCs: =0.96 MES 3 =0.86 MES 2 =0.89 MES 1 Overall accuracy: 77.2% Overall specificity: 85.7% Overall sensitivity: 72.4%	One experienced gastroenterologist and one fellowship physician in gastroenterology
Gutierrez Becker et al. [83]	2021	Retrospective Multi-center	WLE videos from etrolizumab Phase II Eucalyptus and Phase III Hickory and Laurel clinical trials	Quality control model-CNN	1105 UC pts	1672 videos	Grading individual MES in UC	AUROCs: =0.84 MES ≥ 1 =0.85 MES ≥ 2 =0.85 MES ≥ 3	Expert gastroenterologists
Gottlieb et al. [84]	2023	Prospective Multi-center	WLE videos from a phase II trial of mirikizumab	Recurrent neural network	249 UC pts	795 videos	Predicting central reader scores	MES: QWK = 0.844 UCEIS: QWK = 0.855	Expert central readers
Fan et al. [85]	2023	Retrospective Single-center	WLE	ResNet-50	332 UC pts	5875 still images and 20 full-length videos	Scoring full-length intestinal inflammatory activity	Mayo-scored task: Accuracy = 86.5% κ = 0.813 UCEIS-scored task: Vascular pattern: Accuracy = 90.7% κ = 0.822 Erosions and ulcers: Accuracy = 84.6% κ = 0.784 Bleeding: Accuracy = 77.7% κ = 0.702	Four endoscopists with 30, 11, 4, and 6 years of experience
Stidham et al. [86]	2024	Retrospective Single-center	WLE videos from the UNIFI clinical trial	Computer vision analysis that spatially mapped MES to generate the cumulative disease score (CDS)	748 induction and 348 maintenance UC pts	N/R	Quantifying endoscopic severity in UC; CDS vs. MES for differentiating response to ustekinumab vs. placebo	CDS: Lower in ustekinumab vs. placebo at weeks 8 and 44 (*p* < 0.0001) Correlated with MES (*p* < 0.0001) Correlated with clinical components of partial Mayo score (*p* < 0.0001) More sensitive vs. MES to endoscopic differences ustekinumab vs. placebo (Hedges’ g = 0.743 vs. 0.460) Mean CDS differed between neighboring MES levels (*p* < 0.0001) Stratification by pretreatment CDS: Ustekinumab more effective vs. placebo, with increasing effect in severe vs. mild disease (*p* < 0.0001)	N/R
Gutierrez Becker et al. [87]	2024	Retrospective Multi-center	WLE videos from phase III Etrolizumab clinical trials	QC model-V7 platform	1953 UC pts	4326 sigmoidoscopy videos	Evaluating endoscopic severity and disease extent in UC using Ulcerative Colitis Severity Classification and Localized Extent (UC-SCALE)	QWK between UC-SCALE and MCES by central reading: =0.79 full video =0.80 colon section QWK between central and local reading = 0.84 AUROCs for MCES at colon section/video level: =0.87/0.89 all MCES =0.94/0.97 MCES 0 =0.81/0.89 MCES 1 =0.82/0.81 MCES 2 =0.91/0.90 MCES 3 UC-SCALE correlated with calprotectin, C-reactive protein, patient-reported outcomes, physician global assessment and Geboes histologic scores (rs = 0.40–0.55, *p* < 0.0001)	Central and local reading (leading IBD gastroenterologists)
Akiyama et al. [88]	2024	Retrospective Single-center	WLE	EP-0002 function by Fujifilm	100 UC pts	490 images	Assessing colonic tissue oxygen saturation (StO2) for evaluation of clinical, endoscopic, and histologic activity in UC	Rectal StO_2_ correlated with Simple Clinical Colitis Activity Index (*p* < 0.001) Accuracy to predict bowel urgency at 40.5% cut-off: AUROC = 0.74 Median StO_2_ values for Mayo endoscopic subscores 0, 1, 2, and 3 = 52%, 47%, 42%, and 39.5% (significant differences for all pairs) Median StO2 values for UCEIS 0–1, UCEIS 2–4, and UCEIS 5–8 = 50%, 44%, and 39.5% (significant differences for all pairs) Median StO_2_ for Geboes scores 0 to 2 = 49%, significantly higher than histologically active disease (Geboes score ≥ 3) AUROCs for endoscopically and histologically active disease: 0.79 and 0.72 at a colonic StO_2_ cut-off of 45.5%	Three board-certified endoscopists and two board-certified pathologists
Martins et al. [89]	2023	Retrospective Single-center	DAE	XCeption model multi-brand CNN	250 DAE exams	6772 images	Detecting ulcers and erosions in CD	Sensitivity = 88.5% Specificity = 99.7% Accuracy = 98.7% AUPRC = 1.00 CNN processed 293.6 frames per second	Two experienced endoscopists
Xie et al. [90]	2024	Retrospective Single-center	DBE	EfficientNet-B5	628 pts	28,155 small-bowel DBE images	Detecting and objectively assessing small-bowel CD	Accuracy: =96.3% for ulcers =95.7% for non-inflammatory stenosis =96.7% for inflammatory stenosis =87.3% for grading the ulcerated surface =87.8% for grading the size of ulcers =85.2% for ulcer depth	Two experienced endoscopists
Udristoiu et al. [91]	2021	Retrospective Single-center	CLE	DL combined with CNN and long short-term memory (LSTM)	54 UC pts (32 with known active disease, 22 controls)	6205 images	Distinguishing between normal and inflamed colonic mucosa in CD	Normal colonic mucosa: round crypts Inflamed mucosa: irregular crypts and tortuous and dilated blood vessels Accuracy = 95.3% Specificity = 92.78% Sensitivity = 94.6% AUROC= 0.98	N/R

WLE: white-light endoscopy; N/R: not reported; UC: ulcerative colitis; AUROC: area under the receiver operating characteristic; IBD: inflammatory bowel disease; CD: Crohn’s disease; MES: Mayo endoscopic score; QWK: quadratic weighted kappa; VCE: virtual chromoendoscopy; UCEIS: Ulcerative Colitis Endoscopic Index of Severity; CNN: convolutional neural network; DL: deep learning; DAE: device-assisted enteroscopy; DBE: double-balloon enteroscopy; CLE: confocal laser endomicroscopy; CE: capsule endoscopy; and AUPRC: area under precision–recall curve.

**Table 3:** Studies on artificial intelligence-based endoscopy for assessment of the histologic activity of inflammatory bowel disease and the prediction of clinical outcomes.

Study	Year of Publication	Study Design	Endoscopic Technique	Artificial Intelligence Platform	N. of Patients	N. of Images	Study Endpoints	Results	Comparator
Iacucci et al. [72]	2023	Retrospective Multi-center	WLE and VCE videos	ResNet-50	283 UC pts	1090 endoscopic videos (67,280 frames)	Predicting histology and risk of flare	VCE videos: HR RHI ≤ 3: AUROC = 0.83 HR NHI I ≤ 1: AUROC = 0.81 HR PHRI ≤ 1: AUROC = 0.81 WLE videos: HR RHI ≤ 3: AUROC = 0.80 HR NHI I ≤ 1: AUROC = 0.81 HR PHRI ≤ 1: AUROC = 0.79 Stratification of risk of flare similar to physician-assessed endoscopy score	Experienced endoscopists from the PICaSSO group Six expert pathologists
Maeda et al. [99]	2019	Retrospective Single-center	EC	CAD system (EB-01)	187 UC pts	Training: 12,900 EC images Validation: 9935 EC images	Predicting persistent histologic inflammation in UC	Overall test segments: Sensitivity = 74% Specificity = 97% Accuracy = 91% Segments with MES 0–1: Sensitivity = 65% Specificity = 98% Accuracy = 91% Per-patient assessment: Sensitivity = 86% Specificity = 93% Accuracy = 89%	Endoscopists (number and experience N/R) Experienced pathologists
Omori et al. [100]	2024	Retrospective Single-center	WLE ultra-magnifying endoscopy vs. conventional light non-magnifying endoscopy	EndoBRAIN-UC system	52 UC pts	N/R	Diagnosing histologic healing in UC	AI for diagnosis of GS < 3.1: Sensitivity = 74.2% Specificity = 93.8% Accuracy = 77.5% MES 0 for diagnosis of GS < 3.1: Sensitivity = 79.2% Specificity = 90.6% Accuracy = 81.2% AI identified GS < 3.1 in MES 1 (*p* = 0.017)	Three endoscopists
Takenaka et al. [101]	2020	Prospective Single-center	WLE	DNUC (deep neural network for evaluation of UC)	Training: 2012 UC pts Validation: 875 UC pts	Training: 40,758 still images Validation: 4187 still images	Predicting endoscopic and histologic remission	Endoscopic remission: Accuracy = 90.1% κ = 0.798 UCEIS: Interclass correlation coefficient = 0.917 Histologic remission: Accuracy = 92.9% κ = 0.859	Three endoscopists with 11, 13, and 32 years’ experience in IBD-endoscopy Three expert gastrointestinal pathologists
Takenaka et al. [102]	2021	Prospective Single-center	WLE	DNUC (deep neural network for evaluation of UC)	875 UC pts	4187 still images	Predicting UC pts prognosis	Mucosal healing: Sensitivity = 92% Specificity = 91.3% Mucosal healing associated with lower risk of worse prognosis: *p* < 0.001 for hospitalization, colectomy, steroid use, clinical relapse (partial Mayo score ≥ 3, PCR ≥ 3 mg/L, calprotectin ≥ 250 μg/g) HRs of DNUC: For hospitalization = 48.4 For colectomy = 46.4 For steroid use = 10.2 For clinical relapse = 8.8	Three endoscopists with 11, 13, and 32 years’ experience in IBD endoscopy
Takenaka et al. [103]	2022	Prospective Multi-center	WLE videos	DNUC (deep neural network for evaluation of UC)	770 UC pts	Colonoscopy full videos (number N/R)	Real-time detection of UC histologic mucosal inflammation	Histologic inflammation (absence/presence): Accuracy = 81% Histologic remission: Sensitivity = 97.9% Specificity = 94.6% Endoscopic remission: Sensitivity = 81.5% Specificity = 94.7% UCEIS: Interclass correlation coefficient = 0.927	Two central reviewer endoscopists with 12 and 14 years’ experience Two central reading pathologists with 10 and 19 years’ experience
Bossuyt et al. [104]	2020	Prospective Multi-center	WLE with red density (RD) function	CAD RD-based algorithm	29 UC pts, six healthy controls	Number of images N/R	Determining UC endoscopic and histologic activity	RD correlated (*p* < 0.0001) with the following: RHI: r = 0.74 MES subscores: r = 0.76 UCEIS: r = 0.74 Vascular pattern: r = 0.72 Bleeding: r = 0.6 Ulcer: r = 0.61 RD ≤ 60: 96% sensitivity and 80% specificity for histologic remission (AUC = 0.95)	Two groups of two IBD endoscopists (two with >10 years’ experience)
Sinonquel et al. [105]	2023	Retrospective Single-center	WLE with red density (RD) function	CAD RD-based algorithm	39 UC pts from RD pilot study, 6 healthy controls	Number of images N/R	Predicting sustained clinical remission using RD	RD ≥ 65: 71% sensitivity and 63% specificity for long-term clinical remission (mortality, hospitalizations, colectomy, flares, and UC therapy changes) (AUC = 0.68) Low correlation with individual parameters of treatment failure (r = 0.05–0.15, *p* = 0.338–0.729)	N/R
Sinonquel et al. [106]	2024	Prospective Single-center	WLE and SWE	CAD models CNN-based (ResNet-50, VoVNet)	112 UC pts	6926 images	Assessing accuracy of WLE-CAD and SWE-CAD systems for UC histologic activity	SWE-CAD: Sensitivity = 88% (96.4% on section level) Specificity = 71.7% (92.9% on section level) Accuracy = 83.3% (95.2% on section level) WLE-CAD: Sensitivity = 73.9% Specificity = 65.6% Accuracy = 67.5% SWE- vs. WLE-CAD = *p* < 0.005	Number and experience of endoscopists N/R Dedicated gastrointestinal pathologist and fellow
Bossuyt et al. [107]	2021	Prospective Single-center	SWE	CAD model	58 UC pts	113 still images	Automatically evaluating changes in mucosal peri-cryptal vascular structures associated with UC activity (number of bleeding pixels, number of pixels with high density)	CAD histologic remission: Sensitivity = 79% (vs. 95% UCEIS, 98% MES) Specificity = 90% (vs. 69% UCEIS, 61% MES) Accuracy = 86% (vs. 79% UCEIS, 74% MES)	Number and experience of endoscopists N/R
Maeda et al. [108]	2022	Prospective Single-center	EC	Endo-BRAIN-UC	61 UC pts healing group, 74 UC pts active group	44,097 images	Stratifying relapse risk of UC pts in clinical remission	Relapse rate: =28.4% AI active group =4.9% AI healing group (*p* < 0.001) Cumulative probability of being relapse-free: log-rank test *p* < 0.001 Cumulative probability of being relapse-free for MES ≤ 1/MES 1/MES 0: log-rank test *p* = 0.003/=0.006/=0.426 Cumulative probability of being relapse-free for MES 0, MES 1, and MES 2–3: log-rank test *p* = 0.018	Two endoscopists trained on the AI system in at least three UC cases
Kuroki et al. [109]	2024	Prospective Single-center	NBI endoscopy	EB-03 prototype	167 UC pts	8853 images	Diagnosing vascular healing and predicting outcomes in UC	Clinical relapse (partial Mayo score > 2) rate: =23.9% AI vascular active group =3% AI vascular healing group (*p* = 0.01) Vascular active status as the only independent factor associated with clinical relapse: HR = 7.98 (*p* = 0.045) Clinical relapse in MES ≤ 1: AUROC = 0.70 for combination of endoscopic remission and vascular healing vs. 0.65 for endoscopic remission alone	Three endoscopists (expertise N/R but registered)
Ogata et al. [110]	2024	Prospective Single-center	WLE	EB-UC2 prototype	110 UC pts in clinical remission	11,472 images	Predicting clinical relapse during 12-month follow-up	Clinical relapse rate: =24.5% AI-based MES 1 =3.2% AI-based MES 0 *p* = 0.01 =16.2% AI-based MES 0–1 =50% AI-based MES 2–3 *p* = 0.03 Endoscopic remission: Sensitivity = 93.8% Specificity = 77.2% Accuracy = 87.1% Inter/intra-observer reproducibility among non-expert endoscopists: Intra-class correlation coefficient = 0.84–0.86/0.89 (vs. 0.64–0.76/0.76 without AI)	Two expert endoscopists and six non-specialist endoscopists

RHI: Robarts Histopathology Index; NHI: Nancy Histological Index; PHRI: PICaSSO Histologic Remission Index; EC: endocytoscopy; WLE: white-light endoscopy; CE: virtual chromoendoscopy; N/R: not reported; UC: ulcerative colitis; AUROC: area under the receiver operating characteristic; IBD: inflammatory bowel disease; CD: Crohn’s disease; CAD: computer-aided detection; MES: Mayo endoscopic score; UCEIS: Ulcerative Colitis Endoscopic Index of Severity; GS: Geboes score; HR: hazard ratio; SWE: single-wavelength endoscopy; and NBI: narrow-band imaging.

## Data Availability

No new data were created or analyzed in this study. Data sharing is not applicable to this article.

## Abbreviations

The following abbreviations are used in this manuscript:

IBD	Inflammatory bowel disease
CD	Crohn’s disease
UC	Ulcerative colitis
GI	Gastrointestinal
AI	Artificial intelligence
NLP	Natural language processing
ML	Machine learning
DL	Deep learning
ANN	Artificial neural network
CNN	Convolutional neural network
CE	Capsule endoscopy
DAE	Device-assisted enteroscopy
HD	High definition
WLE	White-light endoscopy
DCE	Dye-based chromoendoscopy
VCE	Virtual chromoendoscopy
NBI	Narrow-band imaging
OE	Optical enhancement
BLI/LCI	Blue-light imaging and linked color imaging
CLE	Confocal laser endomicroscopy
EC	Endocytoscopy
PICaSSO	Paddington International virtual ChromoendoScopy ScOre
ECSS	EC system score
MEI	Molecular endoscopic imaging
MES	Mayo endoscopic score
AUROC	Area under the receiver operating curve
Grad-CAMs	Gradient-Weighted Class Activation Maps
UCEIS	Ulcerative Colitis Endoscopic Index of Severity
CAD	Computer-aided detection
AUC	Area under curve
UCEGS	UC Endoscopic Gradation Scale
MCES	Mayo Clinic Endoscopic Subscore
RNN	Recurrent neural network
CDS	Cumulative disease score
UC-SCALE	Ulcerative Colitis Severity Classification and Localized Extent
ADSS	Aggregated Disease Severity Score
GS	Geboes score
IOIBD	International Organization for the Study of Inflammatory Bowel Diseases
DNUC	Deep neural ulcerative colitis
RD	Red density
CRC	Colorectal cancer
EMRs	Electronic medical records
ARC	Automated retrieval console
PHRI	Paddington International virtual ChromoendoScopy ScOre (PICaSSO) Histologic Remission Index
RHI	Robarts Histological Index
NHI	Nancy Histological Index
ECAP	Extent, chronicity, activity, and plus score
